# Gender-specific substance use patterns and associations with individual, family, peer, and school factors in 15-year-old Portuguese adolescents: a latent class regression analysis

**DOI:** 10.1186/s13034-019-0281-4

**Published:** 2019-05-10

**Authors:** João Picoito, Constança Santos, Isabel Loureiro, Pedro Aguiar, Carla Nunes

**Affiliations:** 1 Department of Child and Adolescent Psychiatry, Hospital Pediátrico, Centro Hospitalar e Universitário de Coimbra, Rua Doutor Afonso Romão, 3000-609 Coimbra, Portugal; 2 Escola Nacional de Saúde Pública, Universidade NOVA de Lisboa, Avenida Padre Cruz, 1600-560 Lisbon, Portugal; 3 Department of Pediatrics, Centro Hospitalar Cova da Beira, Quinta do Alvito, 6200-251 Covilhã, Portugal

**Keywords:** Adolescence, Substance use, Gender, Differences, Latent class analysis

## Abstract

**Background:**

Adolescence is a critical period of vulnerability to substance use. Recent research has shown that gender differences in adolescence substance use are complex and in constant flux. The present study aims to investigate gender differences in substance use and initiation patterns in male and female adolescents, and to assess individual, family, peer, and school associated factors of these patterns.

**Methods:**

We applied latent class regression analysis to a Portuguese representative population sample of 1551 15-year-old adolescents, drawn from the 2010 ‘Health Behavior in School-Aged Children’ survey, to characterise different profiles of substance use and initiation for boys and girls, and to identify factors associated with latent class membership, stratifying the associations analysis by gender.

**Results:**

Three common classes were found for both genders, specifically, *Non*-*Users* (boys [B] 34.42%, girls [G] 26.79%), *Alcohol Experimenters* (B 38.79%, G 43.98%) and *Alcohol and Tobacco Frequent Users* (B 21.31%, G 10.36%), with two additional unique classes: *Alcohol Experimenters and Tobacco Users* in girls (18.87%), and *Early Initiation and Poly*-*Substance Users* in boys (5.48%). Poor school satisfaction, bullying, fighting and higher family affluence scale score formed a common core of associated factors of substance use, although we found gender differences in these associations. In girls, but not in boys, family factors were associated with more problematic substance use. Not living with both parents was associated with girl’s *Alcohol and Tobacco Frequent Users* (gATFU) class (OR 3.78 CI 1.18–12.11) and *Alcohol Experimenters and Tobacco Users* (AETU) class (OR 3.22 CI 1.4–7.44). Poor communication with mother was also associated with gATFU class membership (OR 3.82 CI 1.26–11.53) and AETU class (OR 3.66 CI 1.99–6.75). Additionally, a higher psychological symptoms score was associated with gATFU class membership (OR 1.16 CI 1.02–1.31).

**Conclusion:**

Although we found common patterns and associated factors between boys and girls, we report two unique patterns of substance use in boys and girls and specific associations between family, school and peers, and individual factors with these patterns. These findings underscore the need for substance use prevention and health promotion programmes that address potential differences in substance use patterns and associated factors.

**Electronic supplementary material:**

The online version of this article (10.1186/s13034-019-0281-4) contains supplementary material, which is available to authorized users.

## Introduction

Adolescent substance use is an important modifiable risk behaviour, with significant immediate and lasting adverse health and social consequences. In Europe, among 15 to 16-year-old adolescents, 47% have used alcohol and 23% have used tobacco, by the age of 13 or younger [1]. Early initiation of substance use is associated with worse health outcomes and risky behaviours in adulthood [2]. Adolescence is a critical period of psychological, social and cognitive development, as well as a period of increased vulnerability to substance use, delinquency and sexual risk behaviours. Some authors consider that these risky behaviours stem from the interaction between individual and environmental factors such as family, peers and school, and broader social contexts [3, 4].

There are gender differences in adolescent substance use. Epidemiological data have shown that male adolescents have higher rates of substance use than females [5]. However, more recent research show that this gender gap is complex and may even be inverting or narrowing, especially for alcohol use [6, 7]. Therefore, a growing body of research has focused on neurodevelopmental, reward-related behaviour and decision-making differences between the two genders [3]. Although risk factors for substance use are somewhat similar for both genders, there is evidence that gender modifies the effect of social and peer factors on adolescent substance use [4]. Boys and girls differ in both exposure and response to factors, such as family and peer relations, school attachment, academic achievement, victimisation and social neighbourhood [8, 9]. In fact, a review focusing on risk factors influencing drinking progression among adolescents suggests that boys are more vulnerable to substance use because of social factors like higher tolerance, social expectation in use, and higher influence of parental drinking, while girls display higher permeability to parental control [10].

However, although there are several studies in the literature focusing on gender differences in substance use, few studies address the specific patterns of initiation and use simultaneously, or consider a broad set of predictors, including family, school, peers, and individual factors. To address these gaps, we apply latent class regression analysis to a representative population sample of 15-year-old adolescents, stratifying the analysis by gender. Research on unique substance use and initiation patterns, and associated factors in girls and boys, is needed to inform future tailored prevention strategies for adolescent substance use. This poses a continuous challenge, as the dynamics between temporal trends, gender and regional differences are in constant flux.

## Methods

### Participants

This study is a secondary analysis of the 2010 Portuguese ‘Health Behavior in School-Aged Children (HBSC)’ survey. The HBSC study is a World Health Organization collaborative cross-sectional study, conducted every 4 years in a growing number of countries in Europe and North America. The objective of the HBSC study is to increase the understanding of health, lifestyle behaviours and social context of young people aged 11, 13 and 15 years. Further details on this survey, including design, theoretical framework and ethical approval can be found elsewhere [11]. The Portuguese HBSC 2010 sample comprised 4036 school-aged children from 124 randomly selected public schools. This national sample was representative in terms of age and geographic area. For the present study, we focused on 15-year-olds, n = 1553, because the substance use prevalence tends to increase with age and gender differences are more pronounced during late adolescence and adulthood compared with early adolescence [10].

### Measures

All measures were obtained from the 2010 HBSC self-reported questionnaire [12].

Age of initiation was measured for alcohol, tobacco and drunkenness, by self-report. These indicators were assessed by asking ‘At what age did you first drink alcohol (more than a small amount?’, ‘At what age did you first smoke a cigarette (more than one puff)?’, and ‘At what age did you first get drunk?’, respectively. The answer categories were ‘never’, ‘11 years or younger’, ‘12 years’, ‘13 years’, ‘14 years’, ‘15 years’ and ‘16 years or older’. Responses were recoded into never, 13 years or older, and 12 years or younger. Early initiation of substance use is typically defined as being prior to age 13 [13, 14], corresponding roughly to the transition between preadolescence and adolescence. Accordingly, we set the cut-off for early initiation as being before 13 years, in concordance with previous research [14, 15], and yielding additionally sufficient numbers in each group for analyses. Current smoking, alcohol use and drunkenness were assessed by asking ‘On how many occasions (if any) have you done the following things in the last 30 days: smoked cigarettes; drunk alcohol; been dunk?’, respectively. The answer categories were ‘never’, ‘once or twice’, 3–5 times’, ‘6–9 times’, ’10–19 times’, ’20–39 times’, ’40 times or more’.

Lifetime cannabis use was measured asking ‘Have you ever used marijuana (pot, weed, hashish, joint) in your lifetime?’ The answer categories were ‘never’, ‘once or twice’, ‘3–5 times’, ‘6–9 times’, ‘10–19 times’, ‘20–39 times’, ‘40 times or more’.

The selection of family, peer, school and psychosocial factors included in the latent class regression analysis was based on existing literature [16–22] and was already imbedded in the HBSC study survey. Demographic variables included age and gender. Family socioeconomic status was measured with the family affluence scale (FAS) [23], which was constructed with four questions: (1) ‘How many computers does your family own?’, [‘None’ (0), ‘One’ (1), ‘Two’ (2), ‘More than two’ (3)]; (2) ‘Do you have your own bedroom?’, [‘No’ (0), ‘Yes’ (1)]; (3) ‘Does your family own a car, van or truck?’, [‘No’ (0), ‘Yes, one’ (1), ‘Yes, two or more’ (2)]; (4) During the past 12 months, how many times did you travel away on vacation with your family?, [Not at all (0), Once (1), Twice (2), More than twice (3)]. The score of each question was summed, with values ranging from 0 to 9. Family factors included family structure and communication with parents. Family structure was defined as living with both parents and other family structure (as in [20, 24]). Communication with parents was measured separately for the mother and father. These items were evaluated by asking ‘How easy is it for you to talk to the following persons about things that really bother you?’. The answer categories were ‘very easy’, ‘easy’, ‘difficult’, ‘very difficult’, and ‘don’t have or see this person’. Responses were trichotomised into 0 = very easy or easy, 1 = difficult or very difficult, and 2 = don’t have or see (as in [16, 25]).

School factors included perceived school performance and school satisfaction. Perceived school performance is a proxy for academic achievement. Adolescents were asked ‘In your opinion, what does your class teacher(s) think about your school performance compared to your classmates?’. The answer categories were ‘very good’, ‘good’, ‘average’ and ‘below average’. Responses were dichotomised into 0 = very good or good, 1 = average or below average (as in [24]). School satisfaction was measured by asking ‘How do you feel about school at present?’, with the following response categories: ‘I like it a lot’, ‘I like it a bit’, ‘I don’t like it very much’, ‘I don’t like it at all’. Responses were dichotomised into 0 = like it a lot/a bit, and 1 = don’t like it very much/at all (as in [24]).

Peer factors including bullying, victimisation and fighting were also assessed. Bullying was evaluated asking adolescents ‘How often have you taken part in bullying another student(s) at school in the past couple of months?’. Victimisation was assessed asking ‘How often have you been bullied at school in the past couple of months?’. The answer categories were ‘haven’t’, ‘once or twice’, ‘2 or 3 times a month’, ‘about once a week’, and ‘several times a week’. Responses were dichotomised into 0 = never, and 1 = at least once (as in [20, 26]). Fighting was measured by asking ‘During the past 12 months, how many times were you in a physical fight?’, with the following response categories: ‘I have not been’, ‘1 time’, ‘2 times’, ‘3 times’, ‘4 times or more’. Responses were recoded into 0 = never, or 1 = at least once (as in [27]).

Psychological symptoms were measured using a 4-item checklist (Cronbach’s alpha = 0.74), focusing on internalising problems specifically feeling low or depressed, feeling irritable or bad tempered, feeling nervous, and sleeping difficulties, in the past 6 months. The sum score of the four items (range 4–20) was used as a measure of global psychological distress (as in [28]). Physical symptoms were assessed with a 4-item checklist (Cronbach’s alpha = 0.68), encompassing past 6 months report of headache, backache, stomach-ache and dizziness. As with psychological symptoms, the sum score of the four items was used as a measure of somatic/physical complaints (as in [29]).

### Statistical analyses

First, latent class analysis (LCA) was performed to define subgroups of adolescents based on their response patterns on the substance use and initiation indicators. LCA is a common statistical method used in social and behavioural sciences, especially in the fields of addictions and delinquency [30]. It is a type of finite mixture modelling that identifies discrete and mutually exclusive groups (called classes) of individuals within a population [31, 32]. The optimal number of latent classes was determined iteratively, with models ranging from 1 to 7 classes. The best model fit was determined assessing fit criteria, specifically the Bayesian information criterion (BIC), sample-size adjusted BIC (aBIC), Akaike information criterion (AIC), corrected Akaike information criterion (AICC), and Entropy for each model, and considering interpretability and parsimony [33]. The Bootstrap likelihood ratio test (BRLT) was also computed, comparing the model fit between k − 1 and k class models [34]. For BIC, aBIC, AIC, and AICC, smaller values represent better model fit and parsimony. Entropy is a measure of posterior classification uncertainty, measured on a 0 to 1 scale, with values > 0.80 indicating less classification error [34, 35]. For the initial model, we tested if the same class structure applied to boys and girls, comparing a model in which the item-response probabilities were constrained to be equal for both genders, with a model in which the item-response probabilities were allowed to vary. The two models were compared by a standard likelihood-ratio test, as described elsewhere [36]. Following these procedures, a 3-step latent class regression analysis was performed to examine the associations between individual, family, peer and school factors and latent classes, comparing class membership to a reference class. Firstly, the latent class model was estimated only with latent class indicators (substance use and initiation), with the previously determined number of classes. Subsequently, using the latent class posterior probabilities obtained in the first step, the most likely class variable was calculated. In the final step, the most likely class was regressed on predictor variables, adjusting for the classification error [37]. To avoid local maxima, multiple starting values (5000 starts, 1000 optimizations) were used for all models. Additionally, for the latent class regression analysis models, we inspected all solutions to determine if the classes could be distinguished and related to the LCA models without covariates. Furthermore, all analyses accounted for the clustering of students within school classes. The analyses were conducted using Mplus version 8.2 [38] and R version 3.4.3 and 3.5.1, with the LCCA package version 2.0.0 [36].

### Missing data

Of all the cases, 13.3% had missing values for substance use indicators and/or covariates. Each covariate and substance use indicator had less than 5% missing values. Missing values for the substance use indicators were dealt with using full-information maximum likelihood (FIML) procedures, incorporated in the LCA, assuming to be missing at random. However, FIML approaches cannot handle missingness on the predictors of latent class membership [35]. Therefore, we multiply imputed by chained equations 50 datasets for each gender, using the Multiple Imputation by Chained Equation (MICE) package for R. The model of multiple imputation included all covariates used in the latent class regression analysis, as well as the substance use indicators and other variables related to the missing covariates. The 50 datasets for each gender were analysed in Mplus, using the starting values from the first imputation analysis in the subsequent datasets, and pooling results by Rubin’s rules [38, 39]. Two cases had complete missing data on substance use indicators and were listwise deleted. The final sample included 1551 participants. A complete case analysis was also preformed (n = 1346) with similar results.

## Results

### Characteristics of the sample

Tables 1 and 2 report descriptive statistics of adolescents included in this study, including substance use measures and covariates, stratified by gender. In the overall sample, lifetime prevalence for alcohol use was 79.7%, followed by tobacco at 40.4%, and cannabis aat 11.3%.

**Table 1:** Descriptive statistics for sociodemographic, family, school and peer covariates, stratified by gender

Covariates	Boys (n = 680; 43.8%)	Girls (n = 873; 56.2%)	χ^2^/t	*p*
Age	15.46 (0.34)	15.47 (0.34)	− 0.472	0.637
Family Affluence Scale score (range 0–9)	6.04 (1.77)	5.93 (1.86)	1.174	0.241
Family structure			0.338	0.561
Living with both parents	76.8%	78%		
Other	23.2%	22%		
Communication with mother			3.322	0.19
Good	72.2%	72.2%		
Poor	20%	22.8%		
Do not have or see	3.1%	1.9%		
Communication with father			74.985	*< 0.001*
Good	59.4%	38.6%		
Poor	30.4%	51.4%		
Do not have or see	7.1%	7.9%		
Good school satisfaction	70%	79.8%	18.789	*< 0.001*
Good perceived academic achievement	45.3%	42%	1.713	0.191
Bullying (at least once)	35.3%	20.9%	41.201	*< 0.001*
Victimisation (at least once)	37.5%	27.9%	17.209	*< 0.001*
Fighting (at least once)	32.2%	15%	68.732	*< 0.001*
Psychological symptoms^a^ (range 4–20)	7.36 (3.42)	8.885 (3.77)	− 8.2923	*< 0.001*
Somatic symptoms^b^ (range 4–20)	5.78 (2.5)	7.231 (3.36)	− 9.7211	*< 0.001*

Significant values are shown in italics

Results presented in percentage or mean (standard deviation). χ^2^—Chi square test value. t—T test statistic. *p*—p-value

^a^4-item checklist (feeling low or depressed, feeling irritable or bad tempered, feeling nervous, sleeping difficulties in the past 6 months); higher score meaning more symptoms

^b^4-item checklist (headache, backache, stomachache, dizziness, in the past 6 months); higher score meaning more symptoms

**Table 2:** Descriptive statistics for substance use indicators, stratified by gender

Substance use indicator	Boys	Girls	χ^2^	*p*
Alcohol age of initiation			*16.865*	*< 0.001*
Never	18%	20.8%		
≥ 13 years old	29.9%	20.8%		
< 13 years old	52.1%	58.5%		
Drunkenness age of initiation			*6.934*	*0.031*
Never	62.4%	68%		
≥ 13 years old	34%	30%		
< 13 years old	3.4%	2%		
Smoking age of initiation			1.915	0.384
Never	62.4%	58.4%		
≥ 13 years old	34%	33.1%		
< 13 years old	3.4%	7.6%		
Cannabis lifetime			*23.04*	*0.002*
Never	84.4%	90.8%		
Once or twice	6.3%	4.6%		
3–5 times	2.5%	0.8%		
6–9 times	0.9%	0.8%		
10–19 times	1.6%	0.9%		
20–39 times	0.4%	0.6%		
40 times or more	2.9%	0.8%		
Alcohol past 30-day use			*47.962*	*< 0.001*
Never	46.5%	49.9%		
Once or twice	26.9%	35.1%		
3–5 times	12.4%	9.7%		
6–9 times	5.75	2.3%		
10–19 times	4.3%	1.6%		
20–39 times	0.9%	–		
40 times or more	1.5%	0.5%		
Drunkenness past 30-day			11.130	0.133
Never	85%	88.9%		
Once or twice	9.1%	7.9%		
3–5 times	1.9%	0.95		
6–9 times	0.4%	0.5%		
10–19 times	0.6%	0.2%		
20–39 times	0.3%	0.2%		
40 times or more	0.9%	0.1%		
Smoking past 30-day			9.112	0.245
Never	79.4%	80.6%		
Once or twice	6.8%	7.2%		
3–5 times	1.6%	1.8%		
6–9 times	2.8%	1.3%		
10–19 times	2.1%	2.7%		
20–39 times	2.25	2.5%		
40 times or more	4.1%	3.4%		

Significant values are shown in italics

χ^2^—Chi square test value. *p*—p-value

### Model selection

Initially, a 5-class model was identified, including the whole sample (Additional file 1: Table S1). However, this class structure was not appropriate to describe both boys and girls, based on the result of the likelihood-ratio test comparing models with item-response probabilities constrained and not constrained to be equal by gender (p < 0.01). Additionally, inspection of the item-response probabilities by gender for the 5-class model further attested this, with difficult interpretability of the results, especially for the higher-risk classes.

Subsequently, we performed LCA separately for boys and girls (Table 3). For boys, the 4-class solution provided the lowest sample size adjusted BIC and corrected AIC, and the 3-class solution provided the lowest BIC. For girls, the 5-class solution provided the lowest sample size adjusted BIC and corrected AIC, and the 3-class solution provided the lowest BIC. Applying the principle of parsimony [33] and interpretability we ultimately chose the 4-class model for boys and girls, with good entropy (> 0.8) in both models. The bootstrapped likelihood ratio test also supported the better fit of the 4-class solution compared with the 3-class solution for both boys and girls.

**Table 3:** Fit indices for models with different number of latent classes without covariates, for boys and girls separately

Number of classes	Free parameters	LL	AIC	AICC	BIC	aBIC	Entropy	BLRT p-value
Girls								
1	29	− 4557.71	9173.43	9175.49	9311.81	9219.72	NA	NA
2	59	− 4026.9	8171.81	8180.51	8453.35	8265.98	0.836	< 0.001
3	89	− 3883.03	7944.05	7967.51	*8368.75*	8086.11	0.809	< 0.001
4	119	− 3800.78	7839.57	7877.49	8407.43	8029.51	0.807	< 0.001
5	149	− 3739.76	7777.53	*7839.35*	8488.55	*8015.36*	0.835	< 0.001
6	179	− 3699.53	7757.07	7850.06	8611.25	8042.78	0.859	< 0.001
7	209	− *3668.88*	*7755.75*	7888.15	8753.08	8089.35	*0.866*	< 0.001
Boys								
1	30	− 4052.14	8164.28	8167.16	8299.86	8204.61	NA	NA
2	61	− 3560.51	7243.03	7255.3	7518.69	7325.01	0.856	< 0.001
3	92	− 3451.46	7086.93	7116.18	*7502.69*	7210.58	*0.864*	< 0.001
4	123	− 3358.33	6962.67	*7017.73*	7518.52	*7127.99*	0.819	< 0.001
5	154	− 3315.33	6938.66	7029.94	7634.6	7145.64	0.836	< 0.001
6	185	− 3278.94	*6927.88*	7067.76	7763.93	7176.53	0.82	< 0.001
7	216	− *3248.12*	6928.24	7131.59	7904.38	7218.56	0.846	< 0.001

Italic for best values

*LL* log-likelihood, *AIC* Akaike information Criterion, *AICC* Corrected Akaike Information Criterion, *BIC* Bayesian Information Criterion, *aBIC* sample-size adjusted Bayesian Information Criterion, *BLRT* Bootstrapped Likelihood Ratio Test

### Substance use and initiation latent classes

**Fig. 1:**
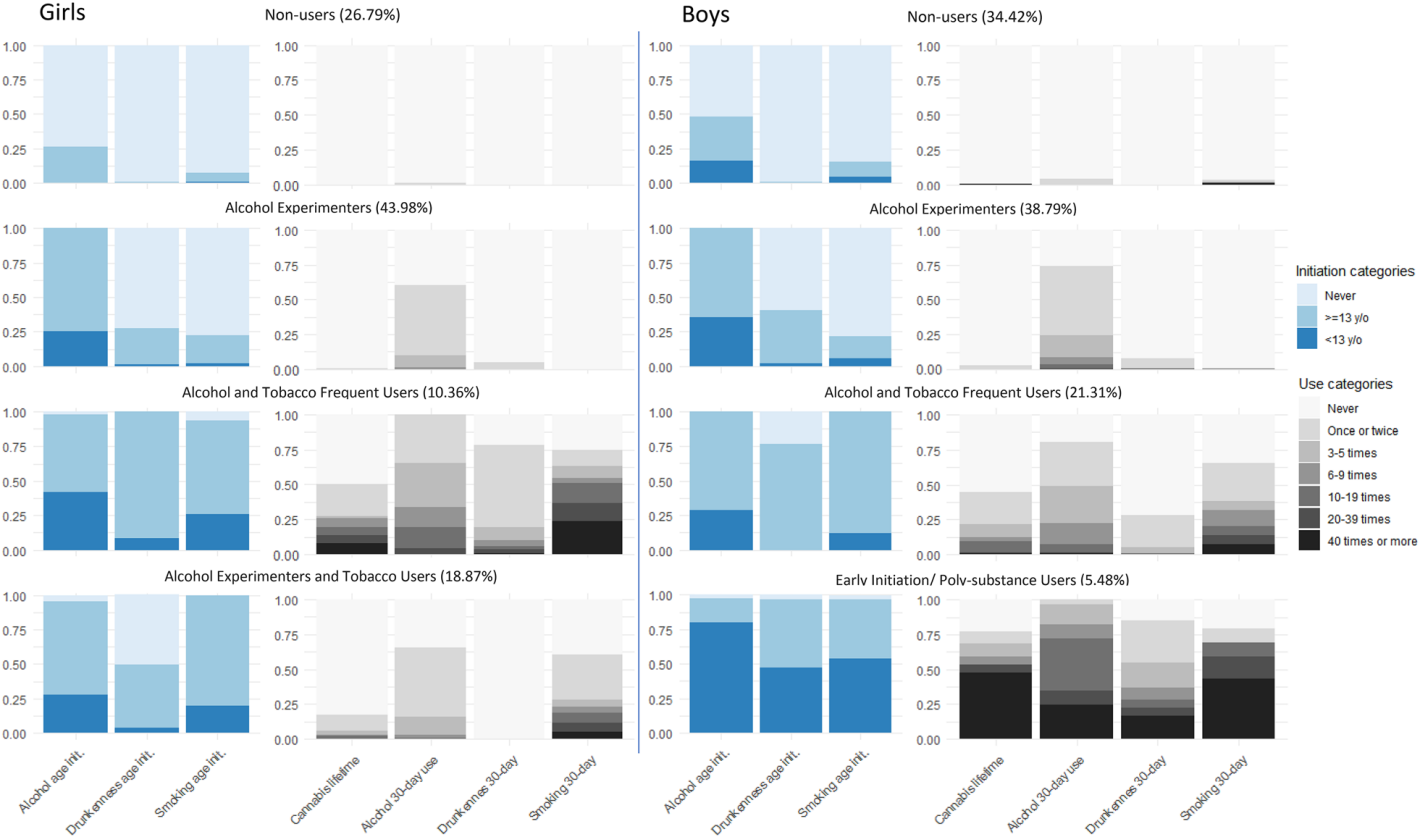
Probability of responses to substance use items conditional on latent class membership. y/o = years old; init. = initiation

Three common classes for both girls and boys were identified, specifically, *Non*-*Users* (36.79%, 34.42%, respectively), *Alcohol Experimenters* (43.98%, 38.79%) and *Alcohol and Tobacco Frequent Users* (10.36%, 21.31%). One unique class was found for girls—*Alcohol Experimenters and Tobacco Users* (18.87%), and another identified for boys—*Early Initiation and Poly*-*Substance Users* (5.48%). Figure 1 shows the estimated class proportions as well as the probabilities of endorsing each item, given class membership, for boys and girls.

*Non*-*Users* had the lowest report of lifetime use and past 30-day use of any substance, with similar results for both boys and girls. *Alcohol Experimenters* was the largest class in both genders, with a higher probability of initiation after age 13 compared with the *Non*-*Users*, but with low past 30-day alcohol use.

*Alcohol and Tobacco Frequent Users* class, for both genders, endorsed high probability of past 30-day alcohol use, smoking, drunkenness, lifetime cannabis use, as well as high probability of early initiation of alcohol, drunkenness and smoking. Girl’s *Alcohol and Tobacco Frequent Users* class tended to present heavier patterns of use, when compared to males’ homonym class. This contrasts with the *Alcohol Experimenters* class in which boys tend to have slightly heavier profiles, when compared to girls.

Girl’s *Alcohol Experimenters and Tobacco Users* are somewhat similar to the *Alcohol Experimenters* class in both boys and girls, but with higher past 30-day smoking, but lower than boy’s and girl’s *Alcohol and Tobacco Frequent Users* and boy’s *Early Initiation and Poly*-*substance Users* class.

Boy’s *Early Initiation and Poly*-*substance Users* class has the highest probability of 40 times or more cannabis lifetime use, and past 30-day alcohol, drunkenness and smoking than any other class in both genders, as well as the highest probability of early initiation.

### Latent class regression analysis

**Fig. 2:**
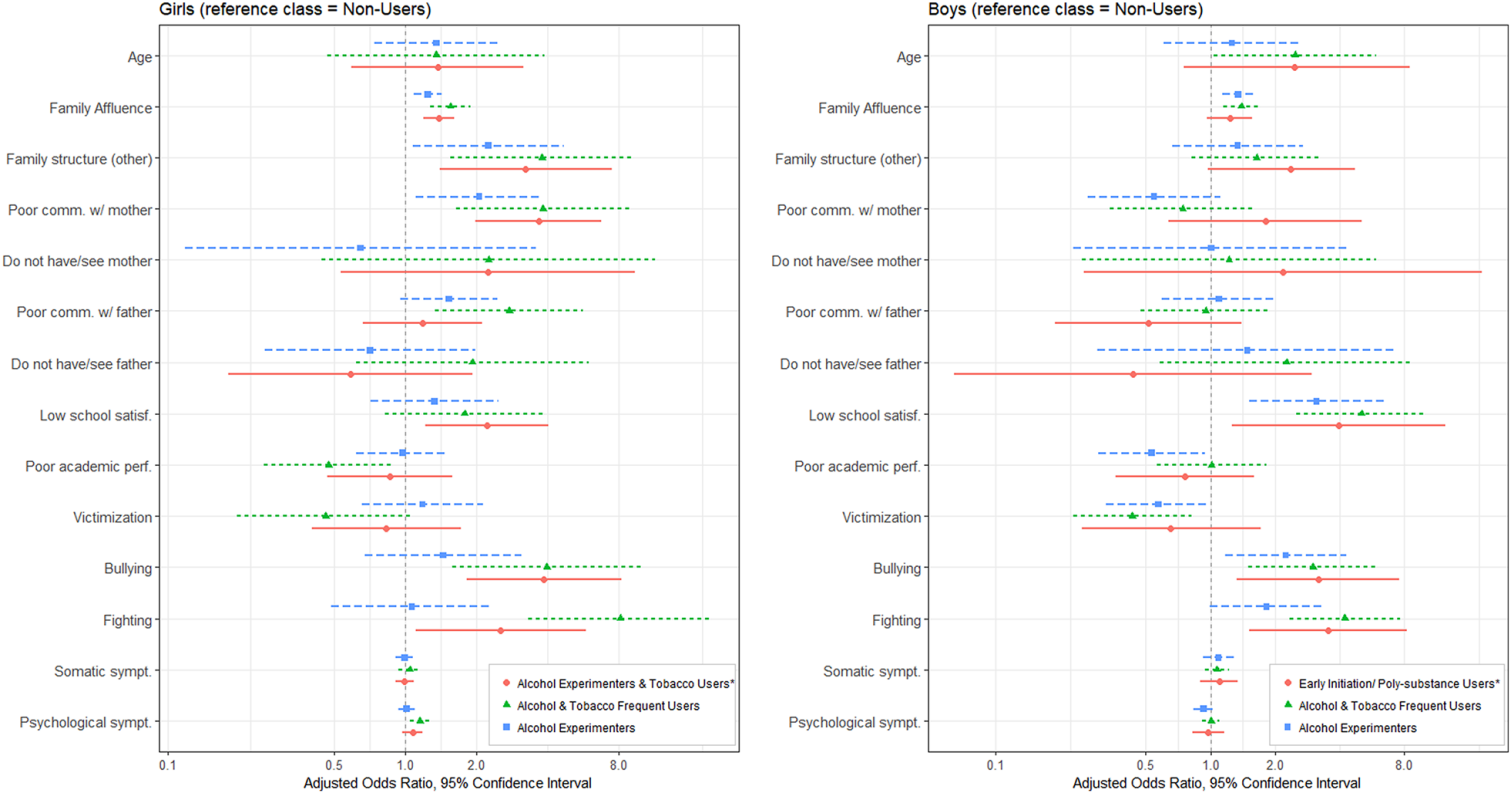
Adjusted odds ratios (full model) between class membership and sociodemographic, family, school and peer factors (reference class *Non*-*Users*). * = unique class; Poor comm. w/mother = poor communication with mother; Poor comm. w/father = poor communication with father

Latent class regression analysis was performed to estimate the adjusted odds ratios between class membership and sociodemographic, family, school, peer and individual factors, stratified by gender (Additional file 1: Table S2 and S3). Figure 2 presents the results with *Non*-*Users* as the reference class. This class was used because it represents the lowest risk class.

#### Alcohol experimenters

Male and female adolescents in the *Alcohol Experimenters* class had higher odds of having higher family affluence score compared to the *Non*-*users* class (odds ratio (OR) 1.33, 95% confidence interval (CI) 1.14–1.61, OR_girls_ 1.25 CI 1.09–1.42). We found gender specific associations, namely family factors for girls, and school and peer factors for boys. Girls in the *Alcohol Experimenters* class have higher odds of not living with both parents (OR_girls_ 2.25 CI 1.08–4.69) and reporting poor communication with their mother (OR_girls_ 2.05 CI 1.11–3.81). Boys present higher odds of low school satisfaction (OR_boys_ 3.12 CI 1.51–6.45) and bullying (OR_boys_ 2.25 CI 4.3), and lower odds of poor perceived academic performance (OR_boys_ 0.53 CI 0.3–0.94), compared with the *Non*-*Users* class.

#### Alcohol and tobacco frequent users

Compared with the *Non*-*users* class, male and female adolescents in the *Alcohol and Tobacco Frequent Users* class had higher odds of involvement in physical fighting and bullying others, with higher odds for girls (bullying OR_boys_ 3.01 CI 1.5–6.01; OR_girls_ 3.97 CI 1.59–9.91; fighting OR_boys_ 4.22 CI 2.33–7.65; OR_girls_ 8.11 CI 2.50–26.29). As for the Alcohol Experimenters class, a higher FAS score was associated with *Alcohol and Tobacco Frequent Users* class membership compared with the *Non*-*Users* class, for both boys and girls (OR_boys_ 1.39 CI 1.09–1.78; OR_girls_ 1.55 CI 1.20–2.02).

However, family factors were specifically associated with *Alcohol and Tobacco Frequent Users* class membership in girls, but not boys, namely not living with both parents (OR_girls_ 3.78 CI 1.56–9.17) and reporting poor communication with their mother (OR_girls_ 3.82 CI 1.64–8.85) and father (OR_girls_ 2.76 CI 1.34–5.65). Additionally, specifically in female adolescents, higher psychological symptoms were associated with higher odds of *Alcohol and Tobacco Frequent Users* class membership (OR_girls_ 1.16 CI 1.05–1.27).

We also found specific associations for Boys in the *Alcohol and Tobacco Frequent Users* class, specifically higher odds of poor school satisfaction (OR_boys_ 5.07 CI 2.52–10.18), and lower odds of victimisation (OR_boys_ 0.43 CI 0.23–0.82), compared with the *Non*-*Users* class.

#### Girl’s alcohol experimenters and tobacco users

This class had similar associations with the *Alcohol and Tobacco Frequent Users* class. Girls not living with both parents (OR_girls_ 3.22 CI 1.4–7.44) as well as girls who reported poor communication with their mother (OR_girls_ 3.66 CI 1.99–6.75) had higher odds of membership to the *Alcohol Experimenters and Tobacco Users* class than the *Non*-*Users* class. School and peer factors such as bullying (OR_girls_ 3.85 CI 1.82–8.17), fighting (OR_girls_ 2.54 CI 1.11–5.8) and poor school satisfaction (OR_girls_ 2.22 CI 2.22–4.04) were associated with higher odds of *Alcohol Experimenters and Tobacco Users* class membership.

#### Boy’s early initiation and poly-substance users

Male adolescents in this class had higher odds of reporting fighting and bullying, comparable to the *Alcohol and Tobacco Frequent Users* class, but with wider confidence intervals (fighting OR_boys_ 3.54 CI 1.52–8.24; bullying OR_boys_ 3.18 CI 1.33–7.59). Contrasting with the other classes in both genders, this class was not associated with higher family affluence score, compared with the *Non*-*Users* class.

We found no associations with class membership for somatic symptoms, and no contact with father or mother figure for any class or gender.

## Discussion

This study demonstrates that there are gender differences in substance use patterns among adolescents, and that both boys and girls can be empirically divided into different subgroups of substance use and initiation. Additionally, we found a common core of associated factors for higher risk substance use patterns among boys and girls, namely higher socioeconomic status, low school satisfaction, bullying and fighting. However, family structure, communication with parents and psychological distress exert different effects according to gender. Female adolescents who report poor parent-adolescent communication and who do not live with both parents have higher odds of belonging to the *Alcohol and Tobacco Frequent Users* class and *Alcohol Experimenters and Tobacco Users* class.

Previous LCA studies [22, 40–42] also reported 4 latent classes of adolescent substance use, spanning from non-users to polysubstance users. A cross-sectional study of 12th grade American adolescents found 6 classes of substance use, with additional profiles such as current smokers and binge drinkers [21]. Some studies also reported a 3-class solution with nonusers, experimenters and multiusers [43, 44]. These results are due to different operationalisation of substance use variables and inclusion of illicit drug use, which makes it difficult to compare substance use classes between studies.

We found more problematic substance use patterns in boys, namely early initiation and poly-substance use. The highest risk profile found in girls (*Alcohol and Tobacco Frequent Users*) was also found in boys, but the profile *Early Initiation and Poly*-*substance Users* was not. Additionally, boys endorsed a higher cannabis lifetime use, especially in the early initiation subgroup. A recent cross-national survey on adolescent substance use [1] reported higher rates of early initiation and frequency of alcohol, tobacco and cannabis use in boys. A longitudinal study focusing on patterns of alcohol use and multiple risk behaviours [45] found that the prevalence of alcohol use at early stages of adolescence was higher in boys, as was higher cannabis use at 15 years. A previous study [46], using data from an ethnically diverse sample of adolescents, also reported that boys were more likely to be polysubstance users, despite the identification of the same class structure for both boys and girls. In a sample of 12th grade American students, females had higher odds of being in the experimenter classes and males in the binge drinker class [21]. However, gender differences in class membership have not been consistently reported in LCA literature, with some studies reporting negative findings [22, 43, 44, 47].

A higher socioeconomic status was associated with riskier substance use classes membership. This result is concordant with previous research [48–50], and may be due to the availability of financial resources that allows easier substance access. However, for the early initiation class, socioeconomic status was not associated with class membership, compared to *Non*-*Users*. A longitudinal study that focused on patterns of cannabis use in adolescence [51] found no association of socioeconomic status with early initiation of cannabis use.

Good school connectedness and satisfaction is associated with better mental health and substance use outcomes [24]. In our study boys who report low school satisfaction had higher odds of membership in all higher risk classes. However, for female adolescents, low school satisfaction was only associated with *Alcohol Experimenters and Tobacco Users* membership. Previous research has consistently associated bullying and physical fighting with substance use and other risk behaviours [52–54]. Correspondingly, boys and girls in the *Alcohol and Tobacco Frequent Users* classes were more likely to report involvement in bullying and fighting, with higher odds for girls compared with boys. Relatedly, in a recent longitudinal study [55], bullying in adolescence is associated with maladjustment and substance use in early adulthood, but only in girls.

Previous research [56, 57] has shown that adolescents living with both biological parents are less likely to engage in illicit or problematic substance use compared with other family typologies. It has been proposed that economic hardship, poorer supervision and parental support, as well as higher levels of negative affect, are responsible for the association of certain family structures (single-parent, stepparent) with adolescent substance use [58]. Our study found that adolescents not living with both parents are more likely to be in higher risk substance use classes, but only for girls. A similar result was reported by a recent cross-national study [57], using data from the 2005/06 HBSC study, in which not living with both parents and having a poor relationship with parents were associated with weekly smoking, especially among girls.

In the literature, family communication has been considered an important protective factor against substance use in adolescence, being a core element of good parenting [59]. Our study found that poor communication with the father and poor communication with the mother were associated with higher odds of membership in risk substance use classes in girls, but not boys. Difficult parent–child communication appears to be a risk factor for low life satisfaction in boys and girls, with easy communication acting as a protective factor only for girls [60]. Previous research has found that female adolescents who lack relational closeness with their fathers are more likely to endorse risk behaviours such as substance use and sexual risk-taking [61]. However, a cross-sectional study of 10th graders who participated in the 2005/06 US HBSC study [16] found that good parental communication was protective for substance use only in boys.

Psychological distress has been associated with adolescent substance use [62]. In our study, a higher psychological symptoms score was associated with *Alcohol and Tobacco Frequent Users* class membership, but only in female adolescents. Accordingly, a recent longitudinal study found bidirectional effects between depressive symptoms and alcohol use, only in girls [63]. Relatedly, using data from a prospective population-based cohort, the association between depressive symptoms and alcohol use was only found for girls [64]. A cross-sectional study of Norwegian high-school students reported the association of higher levels of anxiety symptoms with alcohol consumption only in girls [65].

We did not find any association between somatic symptoms and substance use latent classes membership. In contrast to this result, a cohort study of American 10th grade students reported elevated levels of somatic and depressive symptoms in poly substance users [66]. Similarly, a cluster analysis study of developmental pathways of adolescent substance use found that individuals with a gradual increase in substance use consumption between age 14 and 19 reported more health complaints (headache, backache, stomach ache, tiredness and insomnia) compared with the low use and abstainer group [67].

### Strengths and limitations

LCA has several advantages compared with other alternatives, such as k-means cluster analysis, including probability-based classification, assistance in determination of number of optimal number of clusters, and the possibility for classification and analysis to be performed simultaneously [68]. For the latent class regression analysis, we used the corrected 3-step implemented in Mplus [37], reducing bias in estimates of the strength of association between covariates and latent classes [30, 69]. The sample used is representative of school-aged children in Portuguese public schools and the questionnaire used has good psychometric properties, with several studies showing self-report measures are highly reliable [70]. However, this study is not without limitations. Its cross-sectional design does not allow the establishment of causality. Also, no objective measures of substance use were available. The reliability of the substance use responses could not be controlled, due to no inclusion of a dummy drug in the questionnaire. The study also lacks information about binge drinking or other illicit drugs (cocaine, heroin, ecstasy). The latent classes are dependent on substance use variables operationalisation, and the cut-offs for the categorisation can be somewhat arbitrary; studies that dichotomise substance use indicators may ignore important differences between adolescents who have normative and problematic use [42]. With this issue in mind we preserved the 7-category responses for the substance use indicators. We included different contextual variables, spanning school, peer, and family factors. However, variables on family substance use and attitudes, as well as peer substance use, would be of great relevance to this study.

## Conclusion

This study found three common patterns of substance use in boys and girls, specifically, *Non*-*users*, *Alcohol Experimenters* and *Alcohol and Tobacco Frequent Users*, but also two different unique patterns*: Alcohol Experimenters and Tobacco Frequent Users* in girls, and *Early initiation and Poly*-*substance Users* class in boys. Although poor school satisfaction, bullying, fighting and higher FAS score formed a common core of associated factors of substance use, we found gender differences for these factors. Girls in the *Alcohol and Tobacco Frequent Users* class have higher odds of fighting and bullying compared with their male counterparts. In girls, but not in boys, poor parental communication and not living with both parents were associated with more problematic substance use. Additionally, psychological symptoms were found to be associated with frequent alcohol and tobacco use, but only in girls. These findings underscore the need for substance use prevention and health promotion programmes tailored to female and male adolescents that account for potential different patterns and associated individual, family, school and peer factors.

## Key findings

- We identified distinct substance use and initiation patterns in boys and girls.
- Early initiation and poly-substance use formed a unique pattern, only found in boys.
- Poor school satisfaction, bullying, fighting and higher family affluence scale score were associated with substance use for both genders.
- In girls, poor parent-adolescent communication is associated with higher risk profiles.
- Psychological symptoms were found to be associated with frequent alcohol and tobacco use, only in girls.

## Additional file

**Additional file 1: Table S1.** Fit indices for models with different number of latent classes without covariates, including boys and girls (n = 1551). **Table S2.** Boys; adjusted odds ratios between class membership and sociodemographic, family, school and peer factors. **Table S3.** Girls; adjusted odds ratios between class membership and sociodemographic, family, school and peer factors. **Table S4.** Individual, family, peer and school factors full characterization.

## Data Availability

The dataset supporting the conclusions of this article is available in the HBSC Data Management Center repository [http://hbsc-nesstar.nsd.no/webview/].’

## References

[1] European School Survey Project on Alcohol and Other Drugs (ESPAD) Group (2016). ESPAD report 2015: results from the European school survey project on alcohol and other drugs.

[2] Gray KM, Squeglia LM (2018). Research review: what have we learned about adolescent substance use?. J Child Psychol Psychiatry.

[3] Hammerslag LR, Gulley JM (2016). Sex differences in behavior and neural development and their role in adolescent vulnerability to substance use. Behav Brain Res.

[4] Dir AL, Bell RL, Adams ZW, Hulvershorn LA (2017). Gender differences in risk factors for adolescent binge drinking and implications for intervention and prevention. Front Psychiatry..

[5] Grüne B, Piontek D, Sleczka P, Kraus L, Pogarell O (2017). Drinking location and drinking culture and their association with alcohol use among girls and boys in Europe. J Stud Alcohol Drugs..

[6] Cheng HG, Anthony JC (2017). A new era for drinking? Epidemiological evidence on adolescent male–female differences in drinking incidence in the United States and Europe. Soc Psychiatry Psychiatr Epidemiol.

[7] Keyes KM, Li G, Hasin DS (2011). Birth cohort effects and gender differences in alcohol epidemiology: a review and synthesis. Alcohol Clin Exp Res.

[8] Bright CL, Sacco P, Kolivoski KM, Stapleton LM, Jun H-J, Morris-Compton D (2017). Gender differences in patterns of substance use and delinquency: a latent transition analysis. J Child Adolesc Subst Abuse..

[9] Whaley RB, Hayes-Smith J, Hayes-Smith R (2013). Gendered pathways? Gender, mediating factors, and the gap in boys’ and girls’ substance use. Crime Delinquency..

[10] Schulte MT, Ramo D, Brown SA (2009). Gender Differences in factors influencing alcohol use and drinking progression among adolescents. Clin Psychol Rev..

[11] Santos T, de Matos MG, Simões MC, do Céu Machado M (2016). Contextual factors related to chronic condition in Portuguese adolescents: highlights from the HBSC/WHO study. Psicol Reflex E Crítica..

[12] Currie C, Zanotti C, Morgan A, Currie D, De Looze M, Roberts C, et al., editors. Social determinants of health and well-being among young people. Health Behaviour in School-aged Children (HBSC) study: international report from the 2009/2010 survey. Copenhagen: WHO Regional Office for Europe; 2012. http://www.euro.who.int/__data/assets/pdf_file/0003/163857/Social-determinants-of-health-and-well-being-among-young-people.pdf. Accessed 16 Mar 2019.

[13] Kingston S, Rose M, Cohen-Serrins J, Knight E (2017). A qualitative study of the context of child and adolescent substance use initiation and patterns of use in the first year for early and later initiators. PLoS ONE.

[14] Swahn MH, Bossarte RM (2007). Gender, early alcohol use, and suicide ideation and attempts: findings from the 2005 youth risk behavior survey. J Adolesc Health.

[15] Swahn MH, Bossarte RM, Choquet M, Hassler C, Falissard B, Chau N (2012). Early substance use initiation and suicide ideation and attempts among students in France and the United States. Int J Public Health..

[16] Luk JW, Farhat T, Iannotti RJ, Simons-Morton BG (2010). Parent-child communication and substance use among adolescents: do father and mother communication play a different role for sons and daughters?. Addict Behav.

[17] Evans-Polce R, Lanza S, Maggs J (2016). Heterogeneity of alcohol, tobacco, and other substance use behaviors in U.S. college students: a latent class analysis. Addict Behav.

[18] Tomczyk S, Hanewinkel R, Isensee B (2015). Multiple substance use patterns in adolescents-A multilevel latent class analysis. Drug Alcohol Depend.

[19] Kelly AB, Chan GC, Mason WA, Williams JW (2015). The relationship between psychological distress and adolescent polydrug use. Psychol Addict Behav.

[20] Harakeh Z, de Looze ME, Schrijvers CTM, van Dorsselaer SAFM, Vollebergh WAM (2012). Individual and environmental predictors of health risk behaviours among Dutch adolescents: the HBSC study. Public Health..

[21] Cleveland MJ, Collins LM, Lanza ST, Greenberg MT, Feinberg ME (2010). Does individual risk moderate the effect of contextual-level protective factors? A latent class analysis of substance use. J Prev Interv Community..

[22] Connell CM, Gilreath TD, Hansen NB (2009). A multiprocess latent class analysis of the co-occurrence of substance use and sexual risk behavior among adolescents. J Stud Alcohol Drugs..

[23] Currie C, Molcho M, Boyce W, Holstein B, Torsheim T, Richter M (2008). Researching health inequalities in adolescents: the development of the Health Behaviour in School-Aged Children (HBSC) family affluence scale. Soc Sci Med.

[24] Moore GF, Cox R, Evans RE, Hallingberg B, Hawkins J, Littlecott HJ (2018). School, peer and family relationships and adolescent substance use, subjective wellbeing and mental health symptoms in wales: a cross sectional study. Child Indic Res..

[25] Mark L, Samm A, Tooding L-M, Sisask M, Aasvee K, Zaborskis A (2013). Suicidal ideation, risk factors, and communication with parents. Crisis..

[26] Callaghan M, Kelly C, Molcho M (2019). Bullying and bystander behaviour and health outcomes among adolescents in Ireland. J Epidemiol Community Health.

[27] Djerboua M, Chen BE, Davison CM (2016). Physical fighting, fighting-related injuries and family affluence among Canadian youth. BMC Public Health..

[28] Gariepy G, McKinnon B, Sentenac M, Elgar FJ (2016). Validity and reliability of a brief symptom checklist to measure psychological health in school-aged children. Child Indic Res..

[29] Nuutinen T, Roos E, Ray C, Villberg J, Välimaa R, Rasmussen M (2014). Computer use, sleep duration and health symptoms: a cross-sectional study of 15-year olds in three countries. Int J Public Health..

[30] Feingold A, Tiberio SS, Capaldi DM (2014). New approaches for examining associations with latent categorical variables: applications to substance abuse and aggression. Psychol Addict Behav J Soc Psychol Addict Behav..

[31] Lanza ST, Cooper BR (2016). Latent class analysis for developmental research. Child Dev Perspect..

[32] Schuler MS. Estimating the relative treatment effects of natural clusters of adolescent substance abuse treatment services: Combining latent class analysis and propensity score methods. Ph.D. Thesis. Johns Hopkins University; 2013.

[33] Collins LM, Lanza ST (2013). Latent class and latent transition analysis: with applications in the social, behavioral, and health sciences.

[34] Nylund KL, Asparouhov T, Muthén BO (2007). Deciding on the number of classes in latent class analysis and growth mixture modeling: a monte carlo simulation study. Struct Equ Model Multidiscip J..

[35] Collins LM, Lanza ST (2010). Latent class and latent transition analysis: with applications in the social, behavioral, and health sciences.

[36] Schafer JL, Kang J. LCCA package for R users’ guide (Version 1.1. 0). Retrieved Methodol Psu Edu; 2013.

[37] Asparouhov T, Muthén B (2014). Auxiliary variables in mixture modeling: three-step approaches using M plus. Struct Equ Model Multidiscip J..

[38] Muthen LK, Muthén BO (2017). Mplus version 8 user’s guide.

[39] Rubin DB. Multiple imputation for nonresponse in surveys (Wiley Series in Probability and Statistics); 1987.

[40] Cranford JA, Boyd CJ, McCabe SE (2013). Adolescents’ nonmedical use and excessive medical use of prescription medications and the identification of substance use subgroups. Addict Behav..

[41] Gilreath TD, Astor RA, Estrada JN, Johnson RM, Benbenishty R, Unger JB (2013). Substance use among adolescents in California: a latent class analysis. Subst Use Misuse.

[42] Lamont AE, Woodlief D, Malone PS (2014). Predicting high-risk versus higher-risk substance use during late adolescence from early adolescent risk factors using Latent Class Analysis. Addict Res Theory..

[43] Kelly A, Evans-Whipp TJ, Smith R, Chan GCK, Toumbourou JW, Patton GC (2015). A longitudinal study of the association of adolescent polydrug use, alcohol use, and high school non-completion. Addict Abingdon Engl..

[44] Tomczyk S, Hanewinkel R, Isensee B (2015). Multiple substance use patterns in adolescents—a multilevel latent class analysis. Drug Alcohol Depend.

[45] MacArthur GJ, Smith MC, Melotti R, Heron J, Macleod J, Hickman M (2012). Patterns of alcohol use and multiple risk behaviour by gender during early and late adolescence: the ALSPAC cohort. J Public Health..

[46] Choi HJ, Lu Y, Schulte M, Temple JR (2018). Adolescent substance use: latent class and transition analysis. Addict Behav.

[47] Donovan JE, Molina BSG (2013). Types of alcohol use experience from childhood through adolescence. J Adolesc Health Off Publ Soc Adolesc Med..

[48] Bosque-Prous M, Kuipers MAG, Espelt A, Richter M, Rimpelä A, Perelman J (2017). Adolescent alcohol use and parental and adolescent socioeconomic position in six European cities. BMC Public Health..

[49] Humensky JL (2010). Are adolescents with high socioeconomic status more likely to engage in alcohol and illicit drug use in early adulthood?. Subst Abuse Treat Prev Policy..

[50] Hanson MD, Chen E (2007). Socioeconomic status and substance use behaviors in adolescents: the role of family resources versus family social status. J Health Psychol..

[51] Taylor M, Collin SM, Munafò MR, MacLeod J, Hickman M, Heron J (2017). Patterns of cannabis use during adolescence and their association with harmful substance use behaviour: findings from a UK birth cohort. J Epidemiol Community Health.

[52] Gaete J, Tornero B, Valenzuela D, Rojas-Barahona CA, Salmivalli C, Valenzuela E (2017). Substance use among adolescents involved in bullying: a cross-sectional multilevel study. Front Psychol..

[53] Van Ouytsel J, Torres E, Choi HJ, Ponnet K, Walrave M, Temple JR (2017). The associations between substance use, sexual behaviors, bullying, deviant behaviors, health, and cyber dating abuse perpetration. J Sch Nurs..

[54] Ttofi MM, Farrington DP, Lösel F, Crago RV, Theodorakis N (2016). School bullying and drug use later in life: a meta-analytic investigation. Sch Psychol Q Off J Div Sch Psychol Am Psychol Assoc..

[55] Kretschmer T, Veenstra R, Deković M, Oldehinkel AJ (2017). Bullying development across adolescence, its antecedents, outcomes, and gender-specific patterns. Dev Psychopathol.

[56] Ewing BA, Osilla KC, Pedersen ER, Hunter SB, Miles JN, D’Amico EJ (2015). Longitudinal family effects on substance use among an at-risk adolescent sample. Addict Behav.

[57] Moor I, Rathmann K, Lenzi M, Pförtner T-K, Nagelhout GE, de Looze M (2015). Socioeconomic inequalities in adolescent smoking across 35 countries: a multilevel analysis of the role of family, school and peers. Eur J Public Health..

[58] Hoffmann JP (2017). Family structure and adolescent substance use: an international perspective. Subst Use Misuse.

[59] Carver H, Elliott L, Kennedy C, Hanley J (2017). Parent–child connectedness and communication in relation to alcohol, tobacco and drug use in adolescence: an integrative review of the literature. Drugs Educ Prev Policy..

[60] Levin KA, Dallago L, Currie C (2012). The association between adolescent life satisfaction, family structure, family affluence and gender differences in parent-child communication. Soc Indic Res.

[61] Rostad W. The influence of dad: an investigation of adolescent females’ perceived closeness with fathers and risky behaviors. Master of Arts Thesis. University of Montana; 2012.

[62] Hussong AM, Ennett ST, Cox MJ, Haroon M (2017). A systematic review of the unique prospective association of negative affect symptoms and adolescent substance use controlling for externalizing symptoms. Psychol Addict Behav J Soc Psychol Addict Behav..

[63] Danzo S, Connell AM, Stormshak EA (2017). Associations between alcohol-use and depression symptoms in adolescence: examining gender differences and pathways over time. J Adolesc..

[64] Edwards AC, Joinson C, Dick DM, Kendler KS, Macleod J, Munafò M (2014). The association between depressive symptoms from early to late adolescence and later use and harmful use of alcohol. Eur Child Adolesc Psychiatry.

[65] Johannessen EL, Andersson HW, Bjørngaard JH, Pape K (2017). Anxiety and depression symptoms and alcohol use among adolescents—a cross sectional study of Norwegian secondary school students. BMC Public Health..

[66] Conway KP, Vullo GC, Nichter B, Wang J, Compton WM, Iannotti RJ (2013). Prevalence and patterns of polysubstance use in a nationally representative sample of 10th graders in the United States. J Adolesc Health Off Publ Soc Adolesc Med..

[67] Danielsson A-K, Wennberg P, Tengström A, Romelsjö A (2010). Adolescent alcohol use trajectories: predictors and subsequent problems. Addict Behav.

[68] Magidson J, Vermunt J (2002). Latent class models for clustering: a comparison with K-means. Can J Mark Res..

[69] Bray BC, Lanza ST, Tan X (2015). Eliminating bias in classify-analyze approaches for latent class analysis. Struct Equ Model Multidiscip J..

[70] Roberts C, Freeman J, Samdal O, Schnohr C, Looze M, Nic Gabhainn S (2009). The health behaviour in school-aged children (HBSC) study: methodological developments and current tensions. Int J Public Health..

