# Supplementary material for: Gender-specific substance use patterns and associations with individual, family, peer, and school factors in 15-year-old Portuguese adolescents: a latent class regression analysis
Source: Child Adolesc Psychiatry Ment Health. 2019 May 10;13:21. doi: 10.1186/s13034-019-0281-4 (PMC6511212; doi:10.1186/s13034-019-0281-4)
Supplement: Supplementary file 1 — Additional file 1: Table S1. Fit indices for models with different number of latent classes without covariates, including boys and girls (n = 1551). Table S2. Boys; adjusted odds ratios between class membership and sociodemographic, family, school and peer factors. Table S3. Girls; adjusted odds ratios between class membership and sociodemographic, family, school and peer factors. Table S4. Individual, family, peer and school factors full characterization. [file 13034_2019_281_MOESM1_ESM.docx]

**Gender-specific substance use patterns and associations with individual, family, peer, and school factors in 15-year-old Portuguese adolescents: a latent class regression analysis**

Additional material

| Number of classes | Free parameters | LL | BIC | aBIC | AIC | AICC | Entropy |
| --- | --- | --- | --- | --- | --- | --- | --- |
| 1 | 30 | -8666.348 | 17553.10 | 17457.79 | 17392.70 | 17393.92 | NA |
| 2 | 61 | -7651.866 | 15751.88 | 15558.10 | 15425.73 | 15430.81 | 0.841 |
| 3 | 92 | -7415.145 | 15506.18 | 15213.92 | 15014.29 | 15026.03 | 0.782 |
| 4 | 123 | -7255.926 | 15415.49 | 15024.75 | 14757.85 | 14779.33 | 0.809 |
| 5 | 154 | -7158.017 | 15447.42 | 14958.20 | 14624.03 | 14658.23 | 0.816 |
| 6 | 185 | -7088.779 | 15536.69 | 14948.99 | 14547.56 | 14597.98 | 0.842 |
| 7 | 216 | -7040.663 | 15668.20 | 14982.02 | 14513.33 | 14583.60 | 0.849 |

Table S1 - Fit indices for models with different number of latent classes without covariates, including boys and girls (n=1551)

LL = log-likelihood; BIC = Bayesian information criterion; aBIC = sample size adjusted Bayesian information criterion; AIC = Akaike information criterion; AICC = corrected Akaike information criterion

Table S2 - Boys; adjusted odds ratios between class membership and sociodemographic, family, school and peer factors

| Covariates | Boys - Class membership, Odds Ratio (95% CI) \| Reference class = Non-Users | | | | | |
| --- | --- | --- | --- | --- | --- | --- |
|  | Alc. Experimenters | | Alc. and Tob. Frequent Users | | Very Early Init. / Poly | |
|  | aOR | 95% CI | aOR | 95% CI | aOR | 95% CI |
| **Age** | 1.26 | 0.607-2.618 | 2.481 | 1.04-5.92 | 2.462 | 0.753-8.52 |
| **Family affluence** | **1.352** | **1.138-1.606** | **1.392** | **1.153-1.681** | 1.229 | 0.962-1.569 |
| **Family structure**  **(other)** | 1.339 | 0.666-2.692 | 1.641 | 0.814-3.308 | 2.362 | 0.973-4.737 |
| **Poor communication w/ mother** | 0.546 | 0.268-1.111 | 0.745 | 0.34-1.631 | 1.805 | 0.641-5.082 |
| **Do not have/see mother** | 1.011 | 0.23-4.441 | 1.222 | 0.252-5.93 | 2.175 | 0.258-18.361 |
| **Poor communication w/ father** | 1.095 | 0.592-2.026 | 0.95 | 0.473-1.907 | 0.513 | 0.189-1.398 |
| **Do not have/see father** | 1.487 | 0.299-7.407 | 2.258 | 0.579-8.802 | 0.435 | 0.064-2.956 |
| **Poor aca. perform.** | **0.534** | **0.302-0.942** | 1.013 | 0.562-1.825 | 0.762 | 0.363-1.596 |
| **Low school satisf.** | **3.122** | **1.511-6.449** | 5.067 | 2.521-10.183 | **3.963** | **1.266-12.411** |
| **Bullying** | **2.249** | **1.177-4.296** | **3.006** | **1.504-6.007** | **3.179** | **1.331-7.591** |
| **Victimisation** | **0.572** | **0.328-0.999** | **0.433** | **0.23-0.815** | 0.654 | 0.251-1.711 |
| **Fighting** | 1.826 | 0.995-3.35 | **4.222** | **2.33-7.652** | **3.541** | **1.522-8.24** |
| **Somatic sympt.** | 1.093 | 0.924-1.293 | 1.072 | 0.941-1.221 | 1.098 | 0.901-1.339 |
| **Psychological sympt.** | 0.928 | 0.838-1.027 | 1.006 | 0.916-1.105 | 0.974 | 0.822-1.155 |

Table S3 - Girls; adjusted odds ratios between class membership and sociodemographic, family, school and peer factors

| Covariates | Girls - Class membership, Odds Ratio (95% CI) \| Reference class = Non-Users | | | | | |
| --- | --- | --- | --- | --- | --- | --- |
|  | Alc. Experimenters | | Alc. and Tob. Frequent Users | | Alc. Exp. And Tob. Users | |
|  | aOR | 95% CI | aOR | 95% CI | aOR | 95% CI |
| **Age** | 1.358 | 0.741-2.488 | 1.356 | 0.472-3.893 | 1.372 | 0.594-3.171 |
| **Family affluence** | **1.248** | **1.094-1.424** | **1.554** | **1.272-1.897** | **1.386** | **1.195-1.608** |
| **Family structure**  **(other)** | **2.246** | **1.076-4.692** | **3.782** | **1.56-9.166** | **3.224** | **1.398-7.439** |
| **Poor communication w/ mother** | **2.053** | **1.106-3.812** | **3.815** | **1.644-8.852** | **3.662** | **1.988-6.745** |
| **Do not have/see mother** | 0.649 | 0.118-3.566 | 2.248 | 0.446-11.339 | 2.236 | 0.535-9.339 |
| **Poor communication w/ father** | 1.533 | 0.9582.451 | **2.755** | **1.343-5.649** | 1.183 | 0.665-2.107 |
| **Do not have/see father** | 0.713 | 0.257-1.976 | 1.925 | 0.622-5.957 | 0.589 | 0.179-1.935 |
| **Poor aca. perform.** | 0.978 | 0.625-1.531 | 0.475 | 0.255-0.886 | 0.861 | 0.47-1.577 |
| **Low school satisf.** | 1.335 | 0.717-2.484 | 1.785 | 0.825-3.864 | **2.22** | **1.22-4.042** |
| **Bullying** | 1.445 | 0.674-3.097 | **3.968** | **1.59-9.906** | **3.852** | **1.817-8.168** |
| **Victimisation** | 1.184 | 0.656-2.136 | 0.462 | 0.196-1.09 | 0.833 | 0.405-1.715 |
| **Fighting** | 1.069 | 0.488-2.34 | **8.113** | **3.316-19.849** | **2.537** | **1.111-5.797** |
| **Somatic sympt.** | 0.995 | 0.913-1.084 | 1.046 | 0.937-1.169 | 0.997 | 0.91-1.093 |
| **Psychological sympt.** | 1.018 | 0.941-1.102 | **1.155** | **1.047-1.273** | 1.076 | 0.974-1.189 |

Table S4 – Individual, family, peer and school factors full characterization

| **Individual and context factors** | **Response options** | **Recoded** | **Type of variable** |
| --- | --- | --- | --- |
| **Family factors** |  |  |  |
| **Family Affluence Scale** |  |  | Continuous |
| **Family structure** | Mother, father, stepmother, stepfather, grandmother, grandfather, foster home, other | Living with both parents  Other | Binary |
| **Communication with mother** | Good communication  Mixed communication  Bad communication  Don’t have or see | Good  Poor (mixed+bad)  Don’t have or see | Trichotomic |
| **Communication with father** | (same for communication with mother) | “ | “ |
| **School factors** |  |  |  |
| **Academic Achievement** | “Very good”, “Good”; “Average”, Below average” | Good  Average or below | Binary |
| **School satisfaction** | “Like it a lot, “Like it a bit”, “Don’t like very much”, “Don’t like at all” | Like  Dislike | Binary |
| **Peer factors** |  |  |  |
| **Been bullied last 2 months** | “I haven´t”, “Once or twice”, “2 or 3 times a month”, “once a week”, “several times a week”, | Yes  No | Binary |
| **Bullied others last 2 months** |  |  |  |
| **Participation in fights last 12 months** |  |  |  |
| **Psychological symptoms** |  |  |  |
|  | “In the last 6 months: how often have you had the following…? “About every day” (0), more than once a week” (1), “about every week”(2), “about every month”(3), “rarely or never”(4)   1. Feeling low 2. Irritability or bad temper 3. Feeling nervous 4. Difficulties in getting to sleep | Sum of the four items with score of 0 to 16.  Values recoded for easier interpretability (higher score corresponding to more frequent symptoms). | Continuous |
| **Somatic symptoms** |  |  |  |
|  | “In the last 6 months: how often have you had the following…? “About every day” (0), more than once a week” (1), “about every week”(2), “about every month”(3), “rarely or never”(4)   1. Headache 2. Stomachache 3. Backache 4. Feeling dizzy | Sum of the four items with score of 0 to 16.  Values recoded for easier interpretability (higher score corresponding to more frequent symptoms). | Continuous |
